# Two years of COVID-19 outbreaks in residential care facilities: quantifying workload impact of outbreak control activities on a regional public health team in Ireland, March 2020 to March 2022

**DOI:** 10.1007/s11845-023-03486-4

**Published:** 2023-08-18

**Authors:** Cian Carey, Margaret O’Sullivan, Mary O’Mahony, Anne Sheahan, Peter Barrett

**Affiliations:** 1 Department of Public Health HSE South, St. Finbarr’s Hospital, Cork, Ireland; 2 School of Public Health, University College Cork, Cork, Ireland

**Keywords:** Clinical public health, COVID-19, Outbreak management, Residential care facilities

## Abstract

**Background:**

Ireland, like many countries, pursued a containment strategy during the initial stages of the COVID-19 pandemic. Multidisciplinary Outbreak Control Team (OCT) meetings were among the urgent measures used by public health teams in managing COVID-19 outbreaks, especially in high-risk settings.

**Aim:**

To describe and quantify the resources and person-time involved in managing outbreaks, and conducting OCT meetings, in older person Residential Care Facilities (RCF) in an Irish regional Department of Public Health (DePH) during the first 2 years of the COVID-19 pandemic.

**Methods:**

All COVID-19 RCF outbreaks managed by the DePH HSE-South between March 2020 and March 2022 were identified. Data pertaining to each outbreak, including details of any OCT meetings (frequency, membership, duration) were extracted. Clinical staff members of the DePH were surveyed regarding their time spent on RCF outbreak management.

**Results:**

Two hundred twenty-four outbreaks in older persons RCFs occurred between March 2020 and March 2022 in Cork and Kerry, accounting for 4211 COVID-19 resident/staff cases and 263 resident COVID deaths. One hundred twenty (53.5%) of the outbreaks required at least one OCT meeting, with 374 OCT meetings held in total (range 1–29 meetings per outbreak). Approximately 1819 hours were spent by clinical public health staff on RCF outbreak-related work.

**Conclusions:**

While substantial DePH resources were required to manage COVID-19 outbreaks in older person RCFs, it is highly likely that these efforts prevented new infections within RCFs and thus reduced hospitalisations, ICU admissions and deaths. This sustained input placed a significant burden on the wider multidisciplinary public health team, and it affected the department’s capacity to deal with competing health threats and priorities. Future pandemic preparedness requires commensurate resource planning for public health teams.

## Introduction

COVID-19, caused by the SARS-CoV-2 virus, was first reported in China in December 2019 [1]. Due to the novel nature of this respiratory virus, its disease course was initially uncertain. Early studies reported widespread person-to-person transmission, hospitalisation, severe disease and death, particularly in unvaccinated and highly vulnerable groups such as the elderly and the immunocompromised [2–4].

Ireland, like many countries, pursued a containment strategy during the initial stages of the pandemic, in efforts to contain onward spread of disease. Therefore, substantial public health resources were deployed to contain its spread in high-risk congregate settings such as older person Residential Care Facilities (RCFs). Their residents were often among those at highest risk for severe COVID disease or mortality because of their age profile and medical comorbidities [5]. Multidisciplinary Outbreak Control Team (OCT) meetings were among the urgent measures used in managing COVID-19 outbreaks. OCT meetings can facilitate a coordinated public health response, with the aim of reducing onsite transmission and preventing adverse disease outcomes. However, such meetings are highly resource-intensive, requiring participation of multiple staff members (e.g. specialist doctors, nurses, community support workers, facility management). Several OCT meetings may be required for any given facility, depending on severity and complexity of the outbreak. For public health teams, every decision to proceed with an OCT meeting should be based on a risk assessment, as it invariably entails an opportunity cost, diverting staff time and resources from other public health priorities.

Early COVID-19 guidance in Ireland dictated that OCT meetings should be held for every RCF outbreak with two or more cases which were epidemiologically linked [6]. With the advent of vaccines and improved population-level infection prevention control (IPC) measures, the risk of morbidity and mortality associated with high levels of community transmission of COVID-19 lessened. In the second year of the pandemic, OCT meetings became less important [7].

It is often difficult to quantify the impact of preventive work because much of it is hidden or less tangible than measuring disease incidence [7]. The impact of the COVID-19 pandemic on operational public health teams remains under-researched and arguably, poorly understood. Nonetheless, future pandemic preparedness requires improved understanding and visibility of preventive work, particularly as it pertains to high-risk settings such as RCFs, where the consequences of uncontrolled disease transmission may be severe. We aimed to describe and quantify the resources and person-time involved in managing outbreaks, and conducting OCT meetings, in older person RCFs in a regional Department of Public Health (DePH) during the first 2 years of the COVID-19 pandemic.

## Methods

Under Irish legislation, all new COVID-19 cases and outbreaks necessitated notification to Medical Officers of Health (MOH), based in regional Departments of Public Health [8]. The DePH HSE-South was responsible for coordinating the pandemic response in the Cork and Kerry region of Ireland, which encompasses a diverse urban and rural population of approximately 735,000 people [9].

All clinical details relating to COVID-19 RCF outbreaks managed by the DePH HSE-South were stored on a secure, intra-departmental electronic folder. RCFs were defined as facilities that provided long-term care in a congregate setting. This includes disability, nursing home, mental health, religious and social inclusion services in the region [10]. Both publicly funded and privately funded facilities were included. A 2022 national survey identified 4677 RCF beds in total across counties Cork and Kerry, with an average occupancy rate approaching 90% [11].

We included outbreaks from March 18, 2020 (date of first RCF-related OCT meeting) until March 20, 2022, spanning the first two full years of the pandemic. Eligible RCF outbreaks were identified and relevant data pertaining to each outbreak (including location, frequency of meetings, number of personnel involved) were extracted onto standardised data collection sheets (Appendix 1). The following pandemic periods were used: wave 1 (March–May 2020); summer 2020 (June–August 2020); wave 2 (September–November 2020); wave 3/Alpha (December 2020–March 2021); summer 2021 (April–August 2021); wave 4/Delta (September–November 2021) and wave 5/Omicron (December 2021-March 2022).

All departmental staff members who attended OCT meetings (including doctors and nursing staff, *n* = 18) were surveyed to quantify their estimated working time involved in directly managing RCF outbreaks. The survey inquired about the mean, minimum and maximum duration of OCT meetings, in order to estimate number of hours that staff spent directly engaged in outbreak response meetings (Appendix 2). First and subsequent OCT meetings were assessed separately; the first OCT meeting tends to be longer in duration as the RCF layout needs to be established, and a detailed discussion of staffing is required, as well as comprehensive assessment of known cases, contacts, with initial plans made for implementation of infection, prevention and control (IPC) measures. Additionally, respondents were asked to estimate time spent preparing for, or directly actioning the results of OCT meetings, to better quantify the overall public health staff time involved in outbreak response.

### Statistical analysis

Descriptive statistics were used to summarise the number of RCF outbreaks and related OCT meetings held during the study period. The survey results were grouped and analysed by staff role. The mean, minimum and maximum meeting durations for each group were quantified. The total number of meetings attended by staff were then multiplied by these mean durations. This allowed estimation of overall number of person-hours spent chairing and attending OCT meetings among members of the DePH. Pearson’s coefficient was used to quantify the relationship between the number of OCTs per outbreak and the number of OCT members.

## Results

Two hundred twenty-four outbreaks in older persons RCFs occurred between March 2020 and March 2022 in Cork and Kerry, accounting for 4211 COVID-19 cases (2239 RCF residents and 1972 RCF staff). Overall, 263 COVID-19 deaths were recorded among residents.

One hundred twenty (53.5%) of the outbreaks required at least one OCT meeting. The mean number of OCT meetings held per outbreak was 3 (range 1–29), and in total, 374 OCT meetings were held with RCFs. During wave 1 (March–May 2020), 92% of outbreaks required an OCT meeting, compared with 18% of outbreaks during wave 5. The mean number of attendees (both internal and external to DePH) per OCT meeting was 8.5 (range 2–19), with a moderately strong positive correlation between the number of OCT meetings and the number of attendees at each OCT meeting (*r* = 0.61; *p* < 0.001).

### Survey results

There were 13 respondents to the internal survey (response rate 72%), distributed evenly across medical and nursing grades. The mean duration (and range), total time spent attending OCT meetings and additional time spent on OCT-related work is presented in Table 1. In total, the estimated time spent on OCT-related work was 486 h for consultant doctors, 191 h for other medical staff and 1142 h for nursing staff.

**Table 1:** Estimated time spent attending COVID-19 Outbreak Control Team meetings involving residential care facilities for older persons, Cork & Kerry (March 2020–March 2022)

	**First OCT meeting**		**Subsequent OCT meetings**^f^		**All OCT meetings**	**Additional time**^d^	**Total time**^e^
	*Mean duration*^a^	*Hours*^b^	*Meeting duration*^a^	*Hours*^b^	*Hours*^c^	*Hours*	*Hours*
Consultant (*n* = 4)	48.5 (36–81)	96 (72–162)	31 (22.5–46)	142 (104–210)	238 (176–372)	248	486
SpR/SMO (*n* = 4)	51 (27.5–66)	33 (17–43)	27.5 (19–51)	22 (15–40)	55 (28–82)	136	191
Nursing (*n* = 5)	45 (32.5–66)	110 (79–162)	32.5 (20–40)	174 (106–216)	283 (186–378)	859	1142

Consultant (Consultant in Public Health Medicine, the lead public health doctor and typically chairperson of OCT meeting)

*SpR* Specialist Registrar (in training to become Consultant in the future), *SMO* Senior Medical Officer (career-grade public health doctor with practical expertise in communicable disease prevention and control)

^a^Estimated meeting duration mean and range in minutes, based on OCT meetings attended

^b^Average meeting duration × number of meetings held × number of meeting attendees at that grade

^c^First OCT meeting total hours + subsequent OCT meeting total hours

^d^Calculated from additional time spent on OCT-related work × number of OCT meetings attended by staff at that grade

^e^Sum of all meetings + additional time

^f^Outbreak Control Team (OCT) meetings were separated into whether they were a first/initial meeting, or follow-up OCT meeting for any given outbreak

## Discussion

This study estimates the substantial resources that a regional public health team deployed in managing COVID-19 outbreaks in RCFs, just one of many vulnerable, high-risk community settings and during the first 2 years of the pandemic. We found that approximately 1819 clinical hours (approximately 49 full working weeks of person-time) were needed to manage outbreaks in RCF settings in our region. It is highly likely that these efforts helped to prevent new COVID-19 infections within RCFs and thus hospitalisations, ICU admissions and deaths. It is also likely that outbreak control efforts helped to prevent the health service from becoming overwhelmed during various stages of the pandemic. However, it is important to acknowledge that this constituted only a limited proportion of the department’s overall COVID-19 response work as it does not reflect working time spent addressing other high-priority facilities including acute hospitals, schools, workplaces and social inclusion settings (e.g. migrant accommodation centres, prisons) among many others.

It can be challenging to quantify the clinical workload of operational public health teams and the impact of their preventive work. Furthermore, difficulties with undertaking cost-effectiveness analyses for public health teams have also been described [12]. While these issues are longstanding ones among public health teams, research has shown that they were exacerbated during the pandemic [13]. Reasons for this include the hidden ‘behind the scenes’ nature of public health investigations, variability in data collection systems and inherent sensitivity of data involved. Moreover, the optimal outcome in any outbreak is that chains of transmission should be broken, and new cases of disease cease to arise; both of which may be difficult to quantify. This paper addresses a dearth in the current literature by quantifying the resources needed, using staff time at regional level in Ireland, to manage one of the most significant aspects of the COVID-19 pandemic response. From an operational public health perspective, this information is relevant for future pandemic preparedness and for resourcing of future public health crisis response.

In the initial months of the pandemic, especially in the pre-vaccine phase, OCT meetings are likely to have added substantial value to COVID-19 control efforts, given the high risk of COVID-related morbidity at that time [5]. However, the relative importance of OCT meetings declined with the advent of vaccines and with improved understanding of IPC measures among RCF staff. It is encouraging that only 18% of outbreaks in our region required an OCT meeting following public health risk assessments during wave 5, and this suggests that there may be a lesser requirement for resource-intensive OCTs during future COVID-19 waves if transmissibility and severity of infection remains stable.

Overall, consultants in Public Health Medicine spent approximately 486 h on OCT-related work involving RCFs alone. This equates to over 13 full working weeks, or over a quarter of one working year, in terms of consultant time. While much of this clinical input was warranted, it also placed a significant burden on the wider multidisciplinary public health team, as it affected the capacity of senior public health doctors to lead the response to other competing priorities in our region. The opportunity cost of this cannot be understated, with a recent US study by Kintziger et al. highlighting how COVID prevention work adversely impacted on the ability of the public health workforce to manage other communicable diseases, food-borne illnesses and chronic disease [14].

A key limitation of this study is that we excluded public health investigations for RCFs where only one case was reported on site. While these single case notifications did not qualify as outbreaks and thus did not warrant a full OCT meeting, they often required multiple phone and email consultations with public health staff. This work serves to remind of the challenges involved in quantifying disease prevention work in the absence of a single, national electronic incident management system for all cases and outbreaks of infectious diseases. It is likely that our reported figures underestimate the true person-time required to manage COVID-19 in RCFs in a region of Ireland.

The COVID-19 pandemic has highlighted the importance of having a robust, agile and resilient public health workforce, which can adapt to unanticipated situations. These attributes will be increasingly important moving forward, as public health teams deal with new variants of COVID-19, alongside other emerging health threats such as mpox, novel strains of influenza, other zoonotic illnesses and antimicrobial resistance.

Effective pandemic preparedness requires an acknowledgement and assessment of the operational demands placed on public health teams both now and into the future. Our ability to respond effectively to these new and emerging infectious disease threats will depend on both resources available to public health teams on the ground, and the swift enactment of control measures at the population level.

## Data Availability

The data that support the findings of this study are available from the corresponding author upon reasonable request.

## Appendix 2. Outbreak Control Team (OCT) Meetings Survey

### Survey description

The aim of this project is to describe the workload for one regional Department of Public Health in managing NH outbreaks during the first two years of the COVID-19 pandemic. To achieve this, we require a little bit of information from staff members in the department regarding their time involvement in outbreak control team (OCT) meetings in nursing home facilities. We thank you for taking the time to answer the following few questions pertaining to nursing home OCT meetings.

### List of questions

1. What is your role in the Department of Public Health HSE-South?
  - SPHM
  - SpR
  - SMO
  - Nursing
  - Other (Name)
2. Did you ever attend an OCT meeting?
  - Yes
  - No
3. Would you agree with the following statement: The first OCT meeting for an outbreak tended to be the longest, with further follow up meetings tending to be of shorter duration?
  - Yes (first meeting was longest)
  - No (first meeting was not longer)
  - Unsure
4. Considering the first OCT meeting being held for an outbreak, what was the average amount of time that you would spend attending the meeting & what was the minimum and maximum times that you would have spent attending the first OCT meeting of an outbreak?
  - Average (free text)
  - Min (free text)
  - Max (free text)
5. Considering subsequent (excluding the first) OCT meetings being held for an outbreak, what was the average amount of time that you would spend attending the meeting & what was the minimum and maximum times that you would have spent attending these subsequent OCT meetings?
  - Average (free text)
  - Min (free text)
  - Max (free text)
6. In addition to the time involved in attending an OCT meeting, how much additional time did you spend on average preparing for the meeting and actioning the outcomes of the meeting?
  - Time (free text)
